# Serum Levels of M2BPGi as Short-Term Predictors of Hepatocellular Carcinoma in Untreated Chronic Hepatitis B Patients

**DOI:** 10.1038/s41598-017-14747-5

**Published:** 2017-10-30

**Authors:** Jessica Liu, Hui-Han Hu, Mei-Hsuan Lee, Masaaki Korenaga, Chin-Lan Jen, Richard Batrla-Utermann, Sheng-Nan Lu, Li-Yu Wang, Masashi Mizokami, Chien-Jen Chen, Hwai-I Yang

**Affiliations:** 1 Genomics Research Center, Academia Sinica, Taipei, Taiwan; 2 Institute of Clinical Medicine, National Yang-Ming University, Taipei, Taiwan; 3 The Research Center for Hepatitis and Immunology, National Center for Global Health and Medicine, Ichikawa, Japan; 4 Roche Diagnostics, Ltd, Basel, Switzerland; 5 Department of Gastroenterology, Chang-Gung Memorial Hospital, Kaohsiung, Taiwan; 6 MacKay Medical College, New Taipei City, Taiwan; 7 Graduate Institute of Epidemiology and Preventative Medicine, College of Public Health, National Taiwan University, Taipei, Taiwan

## Abstract

This study examines the role of M2BPGi, a novel seromarker for chronic hepatitis, in predicting hepatocellular carcinoma (HCC) among untreated chronic hepatitis B (CHB) patients. In this nested case-control study, 1070 samples were assayed for M2BPGi, including 357 samples from HCC cases, and 713 samples from non-HCC controls, collected at various times throughout follow-up. HCC case samples were stratified according to years prior to diagnosis. Associations between M2BPGi and HCC were examined with multivariate logistic regression. M2BPGi, α-fetoprotein (AFP), and hepatitis B surface antigen (HBsAg) levels were significant independent short-term predictors of HCC, while M2BPGi was insignificant in long-term analyses. Compared to M2BPGi levels <1.0 cut-off index (COI), those with levels ≥2.0 COI had multivariate odds ratios (95% CI) for HCC of 7.40 (2.40–22.78), 6.46 (2.58–16.18), and 2.24 (0.97–5.15), respectively, for prediction of HCC within 1-2, 2–5, and ≥5 years. Higher proportions of individuals had M2BPGi levels ≥2.0 COI in samples closer to HCC diagnosis. Areas under receiver operating characteristic curves for models with M2BPGi, AFP, and HBsAg levels predicting HCC within 1–2, 2–5, and >5 years were 0.84, 0.81, and 0.75. M2BPGi is a strong and independent short-term predictor of HCC in CHB patients.

## Introduction

Chronic hepatitis B infection (CHB) affects an estimated 400 million people worldwide. The disease often leads to end-stage clinical consequences such as liver cirrhosis and hepatocellular carcinoma (HCC), which are responsible for 0.5 to 1 million deaths per year^1–3^. A large contributor to mortality from CHB is the progression to end-stage liver disease, liver cirrhosis and HCC. Therefore, current treatment strategies aim to improve overall survival and quality of life by preventing the progression to these hard end-points, and by early detection of HCC^2,4–6^.

The disease progression of CHB infection is a dynamic interaction between host, environmental, and virus factors^4,7^. Several predictive risk factors for CHB related cirrhosis and HCC have been well documented in past studies, and include male gender, hepatitis B e antigen (HBeAg) serostatus, increasing age, high serum alanine aminotransferase (ALT) and α-fetoprotein (AFP) levels, alcohol consumption, high serum HBV DNA and hepatitis B surface antigen (HBsAg) levels, family history of HCC, viral genotype, and viral mutants^8–15^. Most of these have been evaluated as long-term predictors of HCC risk, and accurate score-based prediction models have been developed and validated in recent years^10,12,13,16^. Well-established short-term predictors, which are also important for HCC surveillance, are still lacking.

In searching for precursors of HCC development, tracking of liver fibrosis progression and the degree of necroinflammation present are important for determining the severity of liver disease^5,6,17^. Liver biopsy is considered the gold standard for assessment of fibrosis, but is limited by its invasiveness, cost, risk of complications, and by the variable distribution of fibrosis in the liver^5,18^. Therefore, inexpensive, clinically applicable, and noninvasive markers of fibrosis are greatly needed. Previously, several reports had identified M2BP as a new marker for fibrosis in proteome studies^19^. However, recent studies have since reported *Wisteria floribunda* agglutinin-positive human Mac-2-binding protein (WFA + -M2BP) to be a more accurate surrogate glycobiomarker for assessing liver fibrosis in hospital-based treated patients^20^. This marker was later abbreviated to M2BPGi (M2BP glycosylation isomer). In studies of patients with CHB and chronic hepatitis C (HCV), M2BPGi was able to accurately distinguish between stages of fibrosis, with higher levels representing more severe stages of fibrosis^21–26^. In one study from Japan, M2BPGi levels had an area under the receiver operating characteristic curve (AUROC) for diagnosing fibrosis (F ≥ 3) of 0.812, which was comparable or superior to other surrogate fibrosis markers^22^. In addition, M2BPGi levels have also been shown to distinguish stages of fibrosis among Non-alcoholic fatty liver disease (NAFLD) patients^27^. Moreover, M2BPGi levels have also been recently shown to predict incidence of HCC among both HBV and HCV patients, with higher levels associated with higher odds of HCC^20,23–25,28–30^. Interestingly, even under stratification by fibrosis stage, patients with high M2BPGi levels still had higher chances of developing HCC^23^.

To date, major studies of M2BPGi have focused on distinguishing fibrosis, and studies of HCC have been predominantly treated or hospital-based patients, had smaller case numbers, did not parse out prediction of cirrhotic vs. non-cirrhotic HCC, and did not measure different time points prior to HCC to examine trends of M2BPGi. Therefore, the current study aims to examine the role of M2BPGi as a predictor of HCC in a time-dependent manner among a community-based cohort of untreated individuals infected with chronic hepatitis B.

## Methods

### Study Cohort

**Figure 1:**
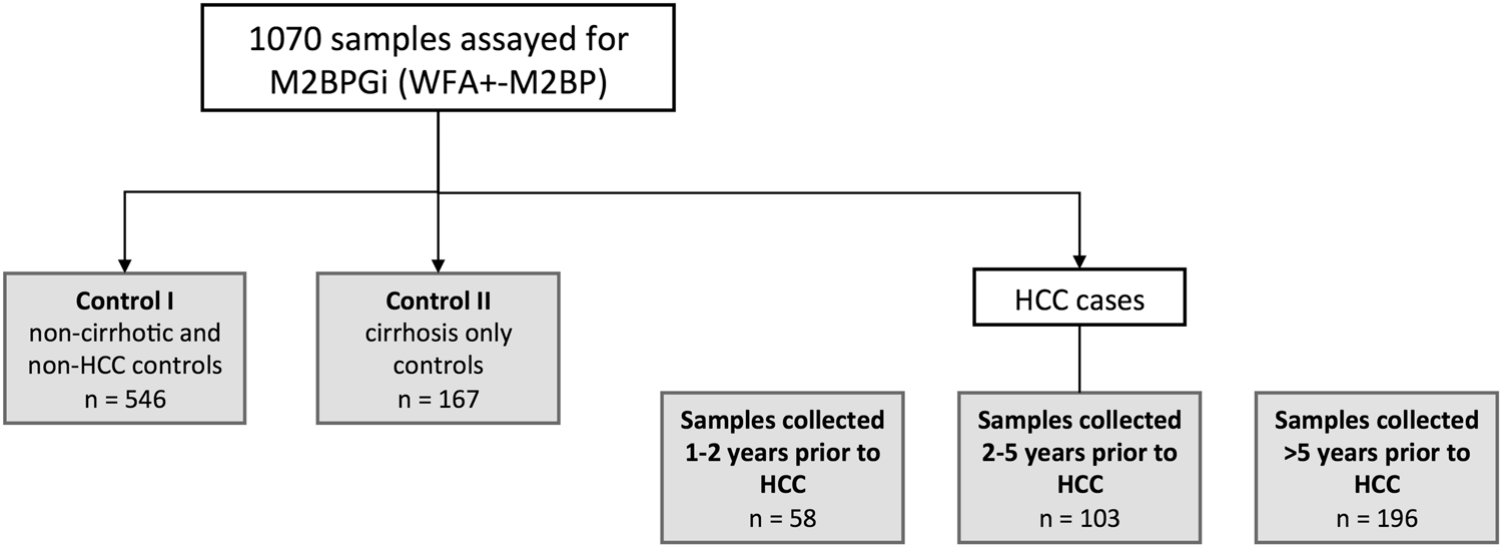
Patient Flow Chart.

Participants were part of the REVEAL-HBV study, a well-documented community-based cohort of untreated individuals. Participants were aged 30–65 years, HBsAg-positive, anti-HCV negative, and free of liver cirrhosis at study entry during 1991–1992. Participants were treatment naïve and followed-up every six to twelve months with examinations that included blood collection for testing of HBV DNA, HBsAg, HBeAg, and ALT, among other markers. Further enrollment and study procedures can be found elsewhere^14,31^. All HCC cases with available pre-diagnosis samples closest to HCC diagnosis were selected for this study. From here, controls were selected at a 1:2 ratio and matched on follow-up age (age when sample was taken) and sex using a nested case-control study design. This group of HCC cases and controls was thus well-matched on age and gender. To examine long-term trajectories of M2BPGi prior to HCC diagnosis or last follow-up, available earlier samples from these cases and controls were also selected. A total of 1070 samples with adequate serum or plasma remaining were assayed for M2BPGi in this study (Fig. 1). Cases included 357 samples from HCC cases, and 713 samples from non-HCC controls, collected at various times throughout follow-up (Fig. 1). The 167 cirrhotic controls were individuals who developed cirrhosis but not HCC during follow-up, and whose samples were selected after cirrhosis diagnosis. Cases were also divided into non-cirrhotic and cirrhotic HCC for analysis.

All participants provided written informed consent. This study was approved by the Institutional Review Board of the College of Public Health, National Taiwan University, Taipei, Taiwan and all study procedures were performed in accordance with the relevant guidelines and regulations on human subjects research.

### Ascertainment of Cirrhosis and HCC

Cirrhosis was diagnosed by high-resolution real-time ultrasonography based on a quantitative scoring system derived from the appearance of liver surface, liver parenchymal texture, intrahepatic blood vessel size, and splenic size. All ultrasonographic examinations were performed and interpreted according to a standardized protocol. To confirm cirrhosis cases, computerized data linkage to the National Health Insurance profiles in Taiwan was performed. Medical records of identified cirrhosis cases were further reviewed by gastroenterologists. Cases of HCC were detected through repeated ultrasound and AFP testing, and computerized linkages with the National Cancer Registry and National Death Certification databases until December 31^st^, 2011. Identified cases were confirmed through chart reviews by gastroenterologists according to the following criteria: histopathologic confirmation; positive lesions detected by at least two different imaging techniques (such as abdominal ultrasonography, angiogram, or computed tomography); or positive lesions detected by one imaging technique combined with a serum α-fetoprotein level greater than 400 ng/mL.

### Laboratory Methods

Blood collections were performed at study entry and follow-ups. Tests on serum markers were performed using commercial kits: HBsAg and HBeAg by radioimmunoassay (Abbott Laboratories, North Chicago, IL), ALT by serum chemistry autoanalyzer (model 736; Hitachi Co., Tokyo, Japan) using commercial reagents, serum HBV DNA levels by polymerase chain reaction (COBAS Amplicor; Roche Diagnostics, Indianapolis, IN) for baseline samples, and by real-time polymerase chain reaction (COBAS TaqMan; Roche Diagnostics, Indianapolis, IN) for follow-up samples. Serum HBsAg levels were quantified using the Elecsys HBsAg II Quant assay (Roche Diagnostics GmbH, Mannheim, Germany). M2BPGi was quantified with a novel sandwich immunoassay using the fully automated chemiluminescence enzyme immunoanalyzer, HISCL-5000 (Sysmex Co., Kobe, Japan)^21^. Raw counts of M2BPGi were converted to a standardized cut-off index (COI) for analysis^32^.

### Statistical Analysis

HBV DNA and HBsAg levels were log (base 10) transformed. Samples collected <1 year prior to HCC diagnosis were excluded, to ensure that no prevalent cases were included. Age refers to the age of the patient when the sample was taken. Values of all seromarkers were measured at the collection point of each sample. Case samples were stratified according to time intervals (1–2, 2–5, 5–7 and >7 years) between sample collection and HCC diagnosis. For multivariable analyses, the longer time intervals were consolidated to >5 years, as results for analyses of 5–7 years and >7 years were essentially equal (not shown). Analyses were performed treating each sample separately, although a limited number of samples from different time points may have come from the same patients. However, this number was few and did not warrant accounting for clustering in analyses. The cutoffs of COI = 1 for M2BPGi was the previously reported cutoff for undetectability^23^. Mantel-Haenszel Chi-Square tests and Pearson correlation was used to examine the linear trend of increasing M2BPGi prior to HCC diagnosis. Using the Youden’s index, COI = 2 was determined as the second cutoff point for analyses. Associations between predictive factors and HCC were assessed with multivariate unconditional logistic regression. Factors significantly associated with both M2BPGi and HCC were included in multivariate models. Predictive accuracy was determined using area under the receiver operating characteristic curves (AUROC). Statistical significance was determined by two-tailed tests (P < 0.05). Statistical analyses were performed with SAS software (version 9.4; SAS Institute, Cary, NC).

## Results

### Cohort Characteristics

The details of 1070 samples examined in overall HCC analyses are shown in Table 1. Cases were stratified according to their sample time intervals. HCC cases had significantly higher serum ALT, aspartate aminotransferase (AST), AFP, HBV DNA, and HBsAg levels, compared to controls. Specifically, AFP levels were higher in HCC cases, particularly in samples collected closest to HCC diagnosis. HCC cases also had significantly higher levels of serum M2BPGi closer to HCC diagnosis.

**Table 1:** Characteristics and distribution of overall cohort (n[%]).

	Controls		HCC cases: stratified by collection time (years prior to HCC diagnosis)				
	Non-LC Controls (n = 546)	LC^6^ Controls (n = 167)	1–2 years (n = 58)	2–5 years (n = 103)	≥5 years (n = 196)	P-value (comparing time points of HCC)	P-value (all HCC cases vs. all controls)
Age (mean years [sd])	61.7 (8.4)	58.8 (8.5)	61.2 (8.8)	59.1 (9.7)	52.5 (9.3)	<0.001	<0.001
Gender							
Female	162 (29.7)	39 (23.4)	11 (19.0)	18 (17.5)	46 (23.5)		
Male	384 (70.3)	128 (76.7)	47 (81.0)	85 (82.5)	150 (76.5)	0.44	0.01
ALT (U/L)^1^							
< 45	477 (90.9)	144 (88.3)	39 (72.2)	58 (63.7)	154 (82.4)		
≥45	48 (9.1)	19 (11.7)	15 (27.8)	33 (36.3)	33 (17.7)	0.003	< 0.001
AST (U/L)^2^							
< 45	497 (94.7)	138 (84.7)	39 (72.2)	56 (61.5)	154 (83.2)		
≥45	28 (5.3)	25 (15.3)	15 (27.8)	35 (38.5)	31 (16.8)	< 0.001	< 0.001
AFP (ng/mL)^3^							
0–10	489 (97.0)	148 (90.8)	31 (54.4)	72 (71.3)	149 (79.3)		
≥10	15 (3.0)	15 (9.2)	26 (45.6)	29 (28.7)	39 (20.7)	< 0.001	< 0.001
HBV DNA level (copies/mL)^4^							
Mean log_10_ copies/mL [sd]	3.4 (3.2)	3.9 (3.1)	4.7 (3.4)	5.5 (2.4)	5.3 (1.9)	0.13	< 0.001
<300	139 (29.1)	35 (21.7)	10 (18.5)	9 (9.6)	14 (7.7)		
300–9999	103 (21.6)	31 (19.3)	5 (9.3)	11 (11.7)	32 (17.6)		
10,000–99,999	75 (15.7)	28 (17.4)	6 (11.1)	11 (11.7)	33 (18.1)		
100,000–999,999	54 (11.3)	19 (11.8)	5 (9.3)	16 (17.0)	27 (14.8)		
≥1,000,000	107 (22.4)	48 (29.8)	28 (51.9)	47 (50.0)	76 (41.8)	0.11	< 0.001
HBsAg (IU/mL)^5^							
Mean log_10_ IU/mL [sd]	1.6 (1.9)	2.2 (1.7)	2.6 (1.6)	2.8 (1.3)	3.0 (1.4)	0.18	< 0.001
<100	240 (45.5)	43 (26.7)	10 (18.9)	17 (17.4)	25 (13.4)		
100–999	145 (27.5)	49 (30.4)	13 (24.5)	26 (26.5)	51 (27.4)		
≥1,000	142 (26.9)	69 (42.9)	30 (56.6)	55 (56.1)	110 (59.1)	0.85	< 0.001
M2BPGi level (index)							
Mean [sd]	0.9 (0.6)	1.9 (3.3)	3.3 (3.9)	2.6 (2.8)	1.2 (0.9)	< 0.001	< 0.001
Negative (C.O.I < 1.00)^7^	339 (62.1)	95 (56.9)	15 (25.9)	34 (33.0)	107 (54.6)		
1.00 < C.O.I < 2.00	181 (33.2)	40 (23.9)	17 (29.3)	30 (29.1)	62 (31.6)		
C.O.I ≥2.00	26 (4.8)	32 (19.2)	26 (44.8)	39 (37.9)	27 (13.8)	< 0.001	< 0.001

^1^Data missing for 50 samples; ^2^Data missing for 52 samples; ^3^Data missing for 57 samples; ^4^Data missing for 95 samples; ^5^Data missing for 45 samples; ^6^LC = cirrhosis. ^7^C.O.I = Cut Off Index.

### M2BPGi and AFP Levels Prior to HCC

**Figure 2:**
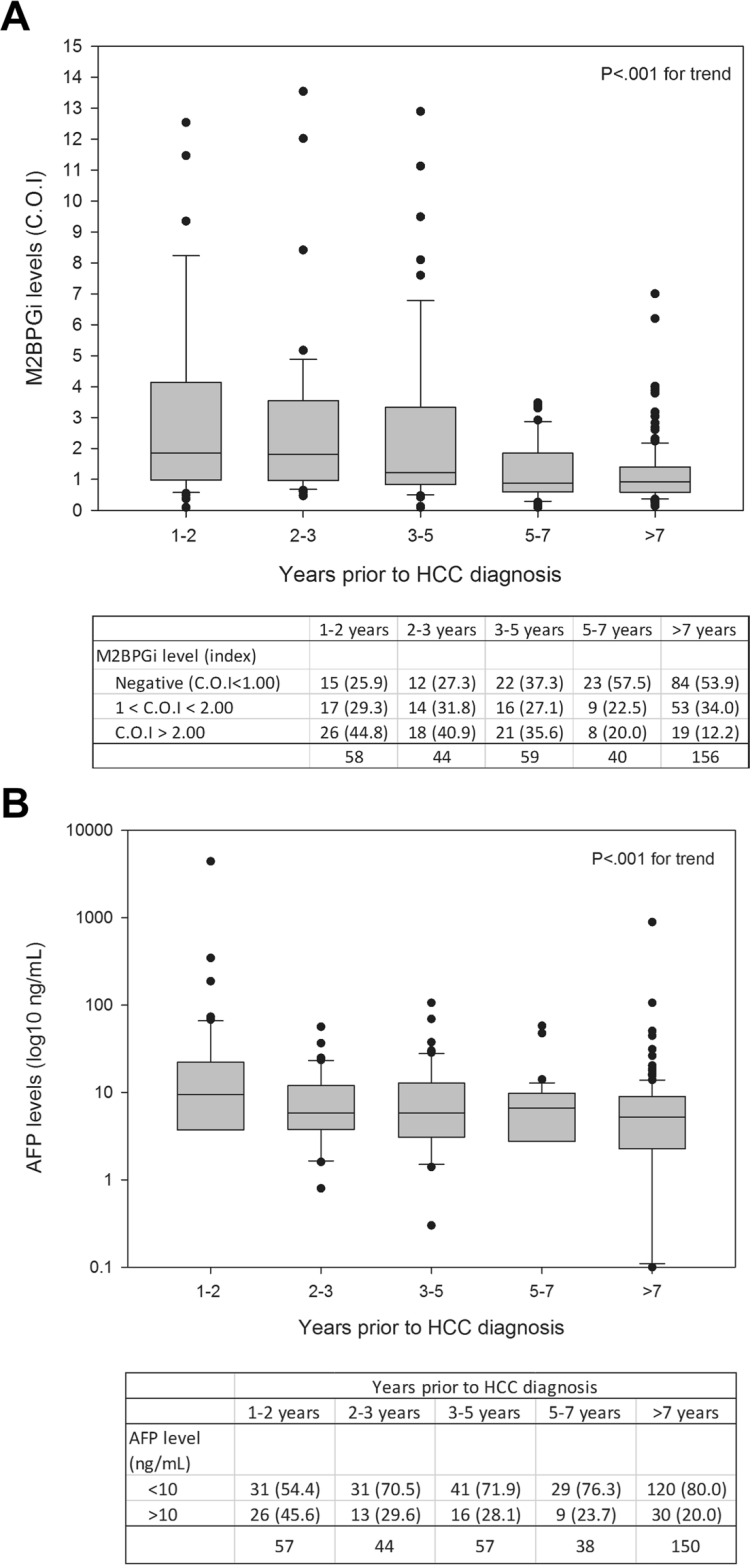
(**A**) Distribution of M2BPGi levels prior to HCC diagnosis; (**B**) Distribution of AFP levels prior to HCC diagnosis.

To investigate time trends of M2BPGi, we examined M2BPGi levels at different time intervals prior to HCC (Fig. 2a). There was an increasing proportion of individuals with M2BPGi levels ≥2.0 COI in samples collected closer to HCC diagnosis. For example, 44.8% of samples collected within 1–2 years of HCC diagnosis and 12.2% of samples collected ≥7 years before HCC diagnosis had M2BPGi levels ≥2.0 COI (P < 0.001 for trend and correlation). A similar trend was seen for AFP, with samples collected closer to HCC diagnosis having higher AFP levels (P < 0.001 for trend, Fig. 2b).

### Prediction of Overall HCC

The ability of M2BPGi to predict short, intermediate, and long-term HCC is shown in Table 2. In multivariate analysis of predicting HCC within two years, only M2BPGi, AFP, and HBsAg levels remained as significant predictors of HCC. Compared to levels < 1.0 COI, those with M2BPGi levels of 1.0–2.0 and ≥2.0 COI had multivariate odds ratios (95% CI) for HCC of 1.64 (0.69–3.91), p = 0.27, and 7.40 (2.40–22.78) p =  < 0.001, respectively. In addition, AFP levels ≥10 ng/mL were associated with a 13-fold increase in risk, while HBsAg levels of 100–999 and ≥1000 IU/mL were associated with a three and four-fold increase in risk, respectively.

**Table 2:** Prediction of overall HCC at three separate time-points (546 controls vs. 357 cases).

	Time between sample collection and HCC diagnosis		
	1–2 years (58 cases)	2–5 years (103 cases)	≥5 years (196 cases)
	Adjusted OR (95% CI)	Adjusted OR (95% CI)	Adjusted OR (95% CI)
M2BPGi level (index)			
Negative (C.O.I < 1.00)	1.00	1.00	1.00
1.00 < C.O.I < 2.00	1.64 (0.69–3.91)	1.31 (0.65–2.63)	1.06 (0.64–1.74)
C.O.I ≥ 2.00	7.40 (2.40–22.78)^c^	6.46 (2.58–16.18)^c^	2.24 (0.97–5.15)
Age (in years)			
30–39	1.00	1.00	1.00
40–49	0.87 (0.25–3.05)	0.50 (0.21–1.19)	0.46 (0.27–0.78)^b^
50–59	0.69 (0.20–2.39)	0.43 (0.18–1.02)	0.22 (0.13–0.39)^c^
≥ 60	1.45 (0.37–5.65)	0.69 (0.24–1.96)	0.05 (0.01–0.20)^c^
Sex			
Female	1.00	1.00	1.00
Male	1.35 (0.50–3.63)	1.54 (0.66–3.58)	1.09 (0.64–1.86)
ALT (U/L)			
<45	1.00	1.00	1.00
≥ 45	1.19 (0.40–3.54)	1.29 (0.58–2.88)	0.92 (0.42–2.01)
AST (U/L)			
< 45	1.00	1.00	1.00
≥ 45	0.77 (0.21–2.83)	3.00 (1.26–7.15)^a^	1.20 (0.50–2.90)
AFP (ng/mL)			
< 10	1.00	1.00	1.00
≥ 10	12.99 (4.82–35.03)^c^	5.40 (2.17–13.45)^c^	5.34 (2.43–11.70)^c^
HBV DNA level (copies/mL)			
< 300	1.00	1.00	1.00
300–9999	0.23 (0.05–1.06)	1.12 (0.34–3.66)	1.34 (0.59–3.06)
10,000–99,999	0.27 (0.06–1.24)	0.96 (0.27–3.49)	1.05 (0.44–2.52)
100,000–999,999	0.22 (0.04–1.27)	2.32 (0.70–7.68)	1.07 (0.42–2.70)
≥ 1,000,000	0.41 (0.10–1.61)	1.22 (0.37–4.06)	1.03 (0.43–2.44)
HBsAg (IU/mL)			
<100	1.00	1.00	1.00
100–999	2.85 (0.79–10.29)	2.16 (0.83–5.64)	3.19 (1.56–6.55)^b^
≥1,000	4.51 (1.21–16.77)^a^	2.77 (1.02–7.53)^a^	5.28 (2.55–10.93)^c^

^a^Indicates significance at the P < 0.05 level (two-tailed test).

^b^Indicates significance at the P < 0.01 level (two-tailed test).

^c^Indicates significance at the P < 0.001 level (two-tailed test).

In multivariate analyses predicting HCC within 2–5 years, results showed M2BPGi, AFP, and HBsAg levels as significant predictors of HCC, while AST levels also showed significance. Compared to M2BPGi levels < 1.0 COI, those with M2BPGi levels ≥2.0 COI had multivariate odds ratios (95% CI) for HCC of 6.46 (2.58–16.18), p < 0.001. Higher AFP levels were associated with a five-fold increase in risk, while HBsAg levels ≥1000 IU/mL were associated with a 2.8-fold increase in risk (Table 2). In predicting HCC ≥ 5 years later, M2BPGi levels could no longer predict HCC. Instead, high AFP levels were associated with a five-fold increase in risk, while HBsAg levels between 100–999 and ≥1000 IU/mL were associated with a three and five-fold increase in HCC risk (Table 2).

Sensitivity analyses comparing HCC cases to all non-HCC controls (consisting of both non-cirrhotic and cirrhotic controls), M2BPGi, AFP, and HBsAg levels remained as the strongest predictors of HCC within 1–2 years. Similarly, M2BPGi predictability decreased with increasing time, while higher AFP and HBsAg levels were again the strongest predictors of HCC at ≥5 years (Supplemental Table 1).

In further long-term analyses using only baseline levels, high AFP levels and increasing HBV DNA levels were significant predictors of HCC, with HBV DNA levels ≥1 million copies/mL associated with an adjusted odds ratio (95% CI) of 7.99 (3.07–20.84) (data not shown).

### Prediction of Cirrhotic and non-Cirrhotic HCC

For non-cirrhotic HCC, M2BPGi was the most significant predictor of non-cirrhotic HCC within 2–5 years, but could not predict non-cirrhotic HCC at ≥5 years (Table 3). Although the adjusted odds ratio was large for prediction within 2 years, small sample sizes did not allow us to see a significant difference. For cirrhotic HCC, however, the pattern was clear. M2BPGi levels were the most significant predictor for cirrhotic HCC within 2 years, with a multivariate odds ratio (95% CI) of 10.07 (2.61–38.86), p < 0.001, for individuals with M2BPGi levels ≥2.0 COI, compared to M2BPGi levels <1.0 COI. The corresponding odds ratios (95% CI) for M2BPGi levels ≥2.0 COI were 7.17 (2.49–20.68), p < 0.001, and 1.98 (0.76–5.18), p = 0.16 for prediction of cirrhotic HCC within 2–5 years and ≥5 years. Other significant predictors included serum AFP levels and in most cases, HBsAg levels (Table 3).

**Table 3:** Prediction of non-cirrhotic and cirrhotic HCC at three separate time-points.

	Non-cirrhotic HCC			Cirrhotic HCC		
	1–2 years^a^ (14 cases)	2–5 years(38 cases)	≥5 years (58 cases)	1–2 years(44 cases)	2–5 years (65 cases)	≥5 years (138 cases)
	Adjusted OR [95% CI]	Adjusted OR [95% CI]	Adjusted OR [95% CI]	Adjusted OR [95% CI]	Adjusted OR [95% CI]	Adjusted OR [95% CI]
M2BPGi level (index)						
Negative (C.O.I < 1.00)	1.00	1.00	1.00	1.00	1.00	1.00
1.00 < C.O.I < 2.00	0.99 (0.27–3.74)	0.89 (0.28–2.83)	0.74 (0.33–1.70)	2.45 (0.77–7.82)	1.65 (0.71–3.79)	1.20 (0.68–2.11)
C.O.I ≥ 2.00	4.65 (0.61–35.50)	6.54 (1.36–31.36)^a^	2.42 (0.73–8.04)	10.07 (2.61–38.86)^c^	7.17 (2.49–20.68)^c^	1.98 (0.76–5.18)
Age (in years)						
30–39	1.00	1.00	1.00	1.00	1.00	1.00
40–49	0.32 (0.04–2.49)	0.12 (0.03–0.54)^b^	0.57 (0.25–1.32)	1.85 (0.39–8.83)	1.17 (0.38–3.58)	0.41 (0.22–0.74)^b^
50–59	0.26 (0.04–1.80)	0.18 (0.05–0.70)^a^	0.34 (0.14–0.82)^b^	1.33 (0.28–6.33)	0.83 (0.26–2.60)	0.17 (0.09–0.33)^c^
≥60	1.68 (0.29–9.71)	0.47 (0.10–2.24)	0.09 (0.01–0.74)^b^	1.29 (0.20–8.33)	1.15 (0.29–4.49)	0.03 (0.01–0.22)^c^
Sex						
Female	1.00	1.00	1.00	1.00	1.00	1.00
Male	0.47 (0.13–1.68)	0.60 (0.17–2.12)	1.32 (0.55–3.18)	2.99 (0.66–13.59)	2.02 (0.69–5.91)	0.89 (0.49–1.64)
ALT (U/L)						
< 45	1.00	1.00	1.00	1.00	1.00	1.00
≥ 45	0.53 (0.03–8.68)	0.75 (0.21–2.72)	0.81 (0.26–2.50)	1.26 (0.38–4.24)	1.35 (0.52–3.54)	1.03 (0.42–2.52)
AST (U/L)						
< 45	1.00	1.00	1.00	1.00	1.00	1.00
≥ 45	2.16 (0.10–46.81)	3.84 (0.98–15.06)	0.45 (0.11–1.95)	0.65 (0.15–2.76)	3.23 (1.16–9.03)^a^	1.29 (0.48–3.48)
AFP (ng/mL)						
<10	1.00	1.00	1.00	1.00	1.00	1.00
≥ 10	7.03 (1.12–44.26)^a^	1.93 (0.36–10.31)	3.10 (0.92–10.44)	12.75 (4.10–39.65)^c^	6.40 (2.41–16.98)^c^	7.03 (3.02–16.37)^c^
HBV DNA level (copies/mL)						
<300		1.00	1.00	1.00	1.00	1.00
300–9999	1.00	0.13 (0.01–1.55)	1.42 (0.36–5.55)	0.64 (0.07–5.70)	2.35 (0.56–9.90)	1.20 (0.45–3.18)
10,000–99,999	0.59 (0.13–2.80)	0.22 (0.02–2.70)	0.60 (0.12–2.95)	0.69 (0.08–6.12)	1.82 (0.40–8.36)	1.20 (0.45–3.25)
100,000–999,999		0.62 (0.08–4.53)	1.00 (0.21–4.80)	0.87 (0.09–8.47)	4.27 (0.97–18.72)	1.06 (0.37–3.06)
≥1,000,000		1.48 (0.22–9.95)	1.33 (0.31–5.79)	0.90 (0.12–6.88)	1.14 (0.25–5.21)	0.88 (0.32–2.39)
HBsAg (IU/mL)						
< 100	1.00	1.00	1.00	1.00	1.00	1.00
100–999	1.07 (0.25–4.58)	6.04 (0.98–37.32)	3.59 (1.04–12.44)^a^	3.86 (0.55–27.29)	1.40 (0.46–4.21)	3.29 (1.41–7.66)^b^
≥1,000	0.72 (0.10–4.92)	2.17 (0.28–16.74)	6.23 (1.77–21.93)^b^	8.86 (1.30–60.46)^a^	2.78 (0.92–8.39)	5.32 (2.29–12.35)^c^

^a^Indicates the time elapsed between sample collection and HCC diagnosis.

^b^Indicates significance at the P < 0.05 level (two-tailed test).

^c^Indicates significance at the P < 0.01 level (two-tailed test).

^d^Indicates significance at the P < 0.001 level (two-tailed test).

In additional analyses of 100 cirrhotic HCC cases and 167 cirrhotic controls who had samples collected after cirrhosis diagnosis, M2BPGi also strongly predicted HCC within 2 years, with multivariate odds ratios (95% CI) of 4.98 (1.10–22.55), p = 0.037, and 8.97 (1.87–43.07), p = 0.006, for M2BPGi levels of 1.0–2.0 and ≥2.0 COI, respectively (Supplemental Table 2). Odds ratios were lower with increasing time, and were non-significant for 2–5 and ≥5 years.

### Prediction Accuracy

The ability of M2BPGi ≥2 COI to predict HCC within 1–2, 2–5, and ≥5 years is shown in Table 4. The sensitivity, specificity, positive predictive value (PPV), and negative predictive value (NPV) to predict HCC within 1–2 years were 45%, 95%, 50%, and 94%, respectively. Specificity remained high (95%) at every time point.

**Table 4:** Prediction of HCC based on M2BPGi ≥2.0.

	Time to HCC Diagnosis		
	1–2 years	2–5 years	≥5 years
Sensitivity	0.45	0.38	0.14
Specificity	0.95	0.95	0.95
Positive predictive value	0.50	0.60	0.51
Negative predictive value	0.94	0.89	0.76

As M2BPGi, AFP levels, and HBsAg levels were frequently significant predictors, a model containing only a combination of these three factors was used to predict HCC (Table 5). AUROC’s for predicting overall HCC within 1–2, 2–5, and ≥5 years were 0.84, 0.81, and 0.75. AUROC’s for models containing M2BPGi, AFP, and HBsAg alone were also computed for comparison (Table 5). Predictive accuracy for M2BPGi alone was significantly higher than AFP or HBsAg alone at short and intermediate intervals, but was decreased for long term prediction. AUROC’s for cirrhotic HCC were significantly higher (P < 0.001) than non-cirrhotic HCC. The highest AUROC’s were seen for prediction within 1–2 years, while prediction of HCC ≥5 years showed the lowest accuracy (Table 5).

**Table 5:** AUROC’s demonstrating predictive accuracy of HCC using M2BPGi, AFP, and qHBsAg.

Years prior to HCC diagnosis	Outcome	Factors included in prediction model			
		Combined M2BPGi, AFP, and qHBsAg	M2BPGi alone	AFP alone	qHBsAg alone
1–2 Years	Overall HCC	0.84	0.79	0.69	0.68
	Cirrhotic HCC	0.92	0.86	0.73	0.75
	Non-cirrhotic HCC	0.68	0.61	0.59	0.53
2–5 Years	Overall HCC	0.81	0.74	0.63	0.68
	Cirrhotic HCC	0.85	0.76	0.66	0.70
	Non-cirrhotic HCC	0.74	0.70	0.59	0.65
>5 Years	Overall HCC	0.75	0.56	0.59	0.70
	Cirrhotic HCC	0.77	0.57	0.60	0.71
	Non-cirrhotic HCC	0.70	0.54	0.56	0.69

## Discussion

This study is the first to use measurements of M2BPGi at various time points prior to HCC to demonstrate the importance of the M2BPGi glycobiomarker as a short-term predictor of HCC in chronic hepatitis B patients. Moreover, this study was conducted in the large cohort of untreated community-based individuals from the REVEAL-HBV study, which includes the advantage of a homogenous protocol-driven prospective follow-up of patients, and allows for the examination of the true role of M2BPGi in the natural history of chronic hepatitis B infection. The major strength of this study is its inclusion of measurements at various time points, allowing for a longitudinal examination of M2BPGi during the course of infection and development of HCC. The large number of HCC cases also allowed for the separate examination of cirrhotic and non-cirrhotic HCC, which was previously not possible.

Previous studies showed increased expression of human M2BP in patients with higher stages of fibrosis. M2BP is a protein that oligomerizes to large “doughnut”-like structures covered with N-glycans^33^. Glycans usually reflect the stage of cell differentiation rather than the level of cellular damage, and a previous study showed changes in N-glycosylation on M2BP during liver disease progression, with increased amounts of altered M2BP detected in higher stages of fibrosis. As WFA specifically binds to the altered N-glycans on M2BP, the rapid assay used in this study accurately quantifies WFA binding M2BP’s (altered M2BP’s). Previous studies of M2BP could only measure total protein expression. In contrast, M2BPGi is a direct measurement of altered M2BPs^19,21^. Moreover, a recent *in vitro* study showed that hepatic stellate cells (HSCs) secreted WFA + -M2BP, which induced Mac-2 expression in Kupffer cells, that in turn activated HSCs to be fibrogenic^34^. Thus, M2BPGi levels should reflect fibrosis progression and not be affected by inflammation or ALT fluctuations. This suggests higher M2BPGi as a useful glycobiomarker reflecting a patient’s proximity to developing HCC^21,32^.

This study found that M2BPGi levels were a strongly significant predictor of HCC, even after adjustment for other traditionally reported risk factors^14^. These results suggest M2BPGi levels as a new non-invasive risk predictor for HCC in chronic hepatitis B patients. Second, this study shows that M2BPGi is an accurate short-term predictor for HCC. Analyses showed higher odds ratios for HCC in samples closer to diagnosis, while long-term analyses of samples collected ≥5 years prior to HCC diagnosis showed no association between M2BPGi and HCC. There were higher proportions of individuals with M2BPGi levels ≥2.0 COI in samples collected closer to HCC diagnosis, while samples collected farther from HCC diagnosis had significantly lower M2BPGi levels (Fig. 2a).

Notably, M2BPGi provides increased predictive accuracy over existing markers such as AFP for short and intermediate term prediction of HCC. While previous studies have shown AFP as a short-term predictor for HCC, we showed that M2BPGi levels alone significantly outperformed both AFP and HBsAg levels in predicting HCC within <2 and 2–5 years. The prediction model including M2BPGi, AFP, and HBsAg levels had AUROC’s as high as 0.84 and 0.92 for predicting overall, and cirrhotic HCC. These results show that HCC patients have significantly increased M2BPGi levels prior to HCC diagnosis. M2BPGi also provided high specificity for ruling out HCC, with very high specificity (95%) across all time points. Furthermore, the correlation between M2BPGi and AFP and HBsAg levels was 0.352 and 0.267, respectively, while the correlation between AFP and HBsAg levels was only 0.172. Thus, these factors are considered independent of each other. Previous studies of short-term HCC (6 years) prediction accurately predicted HCC using multiple serum markers, age, and sex, in addition to HBV seromarkers^35^. However, our study showed that a model using only three seromarkers was able to achieve the same level of accuracy, while also allowing for true short-term prediction (1–2 years) and a non-invasive surrogate assessment of fibrosis.

In this study, HBV DNA levels were only significant in long-term analyses examining baseline levels (not shown). Thus, the significant effect of HBV DNA levels could not be seen in Tables 2 and 3, as analyses were short or intermediate-term. Additionally, the null effect of HBV DNA levels on HCC risk in short term analyses strongly suggests that in the years closer to HCC diagnosis, possibly as necroinflammation and damage have already occurred, viral load no longer further increases HCC risk.

Moreover, recent studies have shown M2BPGi to be an accurate surrogate marker of fibrosis, with comparable or greater accuracy than other surrogate markers^22,26^. Therefore, the ability of M2BPGi to accurately predict cirrhotic HCC can be attributed to its ability identify different fibrotic stages, one of the strongest predictors of HCC development^36^. Studies have also shown that M2BPGi levels provide additional risk stratification, even within each stage of fibrosis. In other words, the significant association between M2BPGi levels and HCC remained, even in non-cirrhotic patients^23,28,29^. The same trend was seen in this study; M2BPGi levels were significant short-term predictors of non-cirrhotic HCC, with moderate accuracy when combined with AFP and HBsAg levels. Although AFP and HBsAg levels did not always reach statistical significance for predicting non-cirrhotic HCC, their addition into the prediction model increased predictive accuracy (Table 4), suggesting that M2BPGi, AFP, and HBsAg provide complementary information for the short-term prediction of non-cirrhotic as well as cirrhotic HCC. Despite these results, the mechanism behind the ability of M2BPGi to provide additional risk stratification still remains unknown.

The availability of M2BPGi in clinical settings may potentially improve management of patients with chronic hepatitis B. Its ability to predict short-term HCC risk will allow better identification of high risk patients who are in need of additional monitoring or immediate initiation of antiviral therapy. Its additional risk stratification can also be incorporated into available risk prediction models to improve the dynamic range of HCC risk prediction, rather than just being a surrogate marker of fibrosis^10,12,16^. Moreover, its non-invasiveness, full automation, utility in both serum and plasma, and high throughput would allow for more efficient management of patients^21^.

There are some limitations to be noted. Detailed fibrosis data was unavailable, as transient elastography was not yet available during the follow-up period, and liver biopsy and expensive marker tests were not feasible in a community-based study of mostly asymptomatic patients. We also could not examine prediction of cirrhosis, as the definition and diagnosis of cirrhosis were not exact in our cohort, due to the aforementioned lack of detailed fibrosis data. This study consisted of untreated individuals infected with genotypes B and C, future studies should examine the role of M2BPGi among treated patients and among those infected with other genotypes.

In conclusion, M2BPGi is a novel independent short-term predictor of HCC in individuals with chronic hepatitis B infection. This study strongly suggests that M2BPGi provides additional information that may improve risk stratification during the clinical management of hepatitis B patients.

## Electronic supplementary material

Supplementary information
