# Supplementary material for: Serum Levels of M2BPGi as Short-Term Predictors of Hepatocellular Carcinoma in Untreated Chronic Hepatitis B Patients
Source: Sci Rep. 2017 Oct 30;7:14352. doi: 10.1038/s41598-017-14747-5 (PMC5662597; doi:10.1038/s41598-017-14747-5)
Supplement: Supplementary file 1 — Supplementary information [file 41598_2017_14747_MOESM1_ESM.pdf]

Supplementary Information

Serum Levels of M2BPGi as Short-Term Predictors of Hepatocellular Carcinoma in Untreated Chronic Hepatitis B Patients

Jessica Liu, PhD[1], Hui-Han Hu, PhD[1], Mei-Hsuan Lee, PhD[2], Masaaki Korenaga, MD[3], Chin-Lan Jen, MSc[1], Richard Batrla-Utermann, MD[4], Sheng-Nan Lu, MD[5], Li-Yu Wang, PhD[6], Masashi Mizokami, MD[3], Chien-Jen Chen, DSc[1,7], Hwai-I Yang, PhD[1,2*]

1. Genomics Research Center, Academia Sinica, Taipei, Taiwan
2. Institute of Clinical Medicine, National Yang-Ming University, Taipei, Taiwan
3. The Research Center for Hepatitis and Immunology, National Center for Global Health and Medicine, Ichikawa, Japan
4. Roche Diagnostics, Ltd, Basel, Switzerland
5. Department of Gastroenterology, Chang-Gung Memorial Hospital, Kaohsiung, Taiwan
6. MacKay Medical College, New Taipei City, Taiwan
7. Graduate Institute of Epidemiology and Preventative Medicine, College of Public Health, National Taiwan University, Taipei, Taiwan

Supplemental Table 1: Prediction of HCC vs. all non-HCC controls (both non-cirrhotic and cirrhotic controls)

	Time between sample collection and HCC diagnosis		
	1-2 years[a] (58 cases)	2-5 years[a] (103 cases)	≥ 5 years[a] (196 cases)
	Adjusted OR (95% CI)	Adjusted OR (95% CI)	Adjusted OR (95% CI)
M2BPGi level (index)			
Negative (C.O.I<1.00)	1.00	1.00	1.00
1.00<C.O.I<2.00	1.92 (0.82-4.47)	1.52 (0.78-2.96)	1.25 (0.78-1.99)
C.O.I≥2.00	4.58 (1.65-12.67)[b]	3.63 (1.62-8.14)[b]	1.18 (0.59-2.36)
Age (in years)			
30 – 39	1.00	1.00	1.00
40 – 49	1.09 (0.33-3.59)	0.67 (0.30-1.50)	0.50 (0.31-0.81)[b]
50 – 59	0.96 (0.30-3.01)	0.48 (0.21-1.08)	0.23 (0.14-0.39)[c]
≥60	2.41 (0.67-8.58)	1.15 (0.44-2.99)	0.06 (0.01-0.24)[c]
Sex			
Female	1.00	1.00	1.00
Male	1.75 (0.69-4.41)	1.50 (0.69-3.25)	0.99 (0.60-1.65)
ALT (U/L)			
<45	1.00	1.00	1.00
≥45	1.75 (0.67-4.53)	2.00 (0.98-4.07)	1.47 (0.73-2.96)
AST (U/L)			
<45	1.00	1.00	1.00
≥45	0.49 (0.16-1.44)	1.69 (0.79-3.63)	0.76 (0.35-1.62)
AFP (ng/mL)			
<10	1.00	1.00	1.00
≥10	8.15 (3.51-18.89)[c]	3.92 (1.87-8.24)[c]	4.00 (2.10-7.63)[c]
HBV DNA level (copies/mL)			
<300	1.00	1.00	1.00
300 - 9999	0.30 (0.07-1.25)	1.33 (0.43-4.12)	1.47 (0.66-3.27)
10,000 - 99,999	0.35 (0.08-1.43)	1.04 (0.30-3.53)	1.05 (0.45-2.44)
100,000 - 999,999	0.26 (0.05-1.32)	2.21 (0.69-7.09)	1.16 (0.48-2.81)
≥1,000,000	0.47 (0.13-1.72)	1.39 (0.45-4.32)	1.06 (0.46-2.42)
HBsAg (IU/mL)			
<100	1.00	1.00	1.00
100 - 999	2.70 (0.78-9.40)[b]	1.91 (0.76-4.79)	2.76 (1.37-5.56)[b]
≥1,000	4.47 (1.28-15.66)[c]	2.21 (0.85-5.75)	4.30 (2.13-8.68)[c]

a. Indicates the time elapsed between sample collection and HCC diagnosis
b. Indicates significance at the P<0.05 level (two-tailed test)
c. Indicates significance at the P<0.01 level (two-tailed test)
d. Indicates significance at the P<.001 level (two-tailed test)

Supplemental Table 2: Prediction of HCC in individuals with cirrhosis

	Cirrhotic HCC		
	1-2 years[a] (32 cases)	2-5 years[a] (49 cases)	≥ 5 years[a] (19 cases)
	Adjusted OR (95% CI)	Adjusted OR (95% CI)	Adjusted OR (95% CI)
M2BPGi level (index)			
Negative (C.O.I<1.00)	1.00	1.00	1.00
1.00<C.O.I<2.00	4.98 (1.10-22.55)[a]	1.71 (0.57-5.10)	1.48 (0.42-5.26)
C.O.I≥2.00	8.97 (1.87-43.07)[b]	2.56 (0.75-8.82)	0.89 (0.16-5.02)
Age (in years)			
30 – 39	1.00	1.00	1.00
40 – 49	2.14 (0.32-14.23)	1.17 (0.31-4.51)	2.22 (0.34-14.61)
50 – 59	2.22 (0.36-13.56)	0.74 (0.19-2.94)	2.65 (0.44-15.86)
≥60	4.83 (0.52-44.60)	5.53 (0.99-31.00)	1.82 (0.11-29.22)
Sex			
Female	1.00	1.00	1.00
Male	15.99 (1.51-169.09)[a]	2.31 (0.58-9.18)	1.62 (0.29-9.04)
ALT (U/L)			
<45	1.00	1.00	1.00
≥45	1.35 (0.28-6.45)	5.05 (1.46-17.48)[a]	1.83 (0.28-11.72)
AST (U/L)			
<45	1.00	1.00	1.00
≥45	0.36 (0.08-1.57)	0.75 (0.22-2.58)	0.69 (0.10-4.67)
AFP (ng/mL)			
<10	1.00	1.00	1.00
≥10	5.60 (1.64-19.13)[b]	3.04 (1.02-9.12)[a]	2.78 (0.66-11.71)
HBV DNA level (copies/mL)			
<300	1.00	1.00	1.00
300 - 9999	0.60 (0.04-8.44)	8.91 (1.12-70.83)[a]	1.22 (0.16-9.20)
10,000 - 99,999	1.65 (0.15-18.61)	3.46 (0.32-37.64)	3.76 (0.48-29.52)
100,000 - 999,999	0.49 (0.04-6.52)	16.12 (1.71-151.90)[a]	4.70 (0.40-55.54)
≥1,000,000	1.28 (0.13-12.57)	5.68 (0.70-45.89)	2.92 (0.30-27.97)
HBsAg (IU/mL)			
<100	1.00	1.00	1.00
100 - 999	2.88 (0.29-28.82)	0.74 (0.18-3.09)	0.30 (0.05-1.70)
≥1,000	5.37 (0.55-52.23)	1.15 (0.29-4.60)	0.34 (0.06-1.99)

a. Indicates the time elapsed between sample collection and HCC diagnosis
b. Indicates significance at the P<0.05 level (two-tailed test)
c. Indicates significance at the P<0.01 level (two-tailed test)
d. Indicates significance at the P<.001 level (two-tailed test)
